# Objectively measured physical activity levels and sedentary time in 7–9-year-old Estonian schoolchildren: independent associations with body composition parameters

**DOI:** 10.1186/s12889-016-3000-6

**Published:** 2016-04-18

**Authors:** Eva-Maria Riso, Merike Kull, Kerli Mooses, Aave Hannus, Jaak Jürimäe

**Affiliations:** Institute of Sports Sciences and Physiotherapy, University of Tartu, 5 Jakobi St, Tartu, 51014 Estonia

**Keywords:** Physical activity, Sedentary time, Body composition, Adiposity indices

## Abstract

**Background:**

Sufficient daily physical activity (PA) is necessary for physical, social and mental health benefits during growth. Most of the available data on children is based on subjective reports, while only limited data on objective PA and sedentary levels is available for primary school children. Increased PA is also an important health indicator of body composition parameters, especially body adiposity indices. The aim of the present study was to determine objectively the amount of daily PA levels at different intensities and sedentary time in normal-weight (NW) and overweight (OW) 7–9-year-old boys and girls, and to find associations between objectively measured PA levels and sedentary time with different body composition values.

**Methods:**

Two hundred and seventy eight (142 boys and 136 girls) primary school children aged 7.9 ± 0.7 years participated in this study. Objective PA intensity and sedentary levels were measured over 7 days by accelerometry. Indices of total fat mass (body fat %, sum of skinfolds), fat distribution (waist-to-height ratio) and muscular component (fat free mass [FFM]) were calculated from measured anthropometric parameters.

**Results:**

There were no differences (*p* > 0.05) in PA intensity levels and sedentary time between boys and girls as well as between NW and OW children. About 11 % of children met the current guidelines of at least 60 min per day of moderate-to-vigorous PA (MVPA). Sedentary time was positively and negatively associated (*p* < 0.05) with all body fat and FFM values, respectively. Moderate and vigorous PA along with MVPA were negatively and positively associated (*p* < 0.05) with all body fat and FFM indices, respectively.

**Conclusions:**

The results of present study showed that about 11 % of primary school children were engaged in PA of at least 60 min of MVPA daily. While MVPA is negatively associated with fat mass indices and positively associated with FFM regardless of different confounders, sedentary time is negatively related to FFM and positively with fat mass values after adjusting for several confounders. These results suggest that higher MVPA level and lower sedentary time level are important in maintaining and developing healthy body composition in primary school children during growth.

## Background

It is widely demonstrated that sufficient physical activity (PA) during childhood has beneficial effects on both short- and long-term health outcomes, and decreases risk factors of several chronic diseases [1, 2]. Despite the well-established physical, social and mental health benefits of regular PA, a substantial proportion of children and adolescents are still not active enough to benefit their health [3]. Insufficient PA has been associated with an increased risk of obesity [1, 2] and with comorbidities such as metabolic or cardiovascular diseases [4, 5]. Accordingly, it is important that children were regularly physically active already at very young age.

The World Health Organization recommends that 6–17 years old children participate in at least 60 min of moderate-to-vigorous physical activity (MVPA) per day [6]. Studies based on self-report questionnaires demonstrate that less than 35 % of children and adolescents follow these recommendations [7]. However, the accuracy of self-reports remains questionable in children and therefore accelerometers have been recommended to assess PA patterns in children objectively [7, 8]. Data from objectively measured PA studies suggest that the PA level of primary school children is not sufficient [5, 9–12]. For example, 43 % of 9–11-year-old Canadian children had an average of 60 min or more MVPA per day [12], while 69 % of 9–10-year-old British children achieved 60 min or more MVPA per day [13]. In the USA, 42 % of 6–11-year-old children obtain the recommended 60 min per day of PA [14]. Among a heterogeneous group of 2–10-year-old Estonian children, 26.8 % boys and 13 % girls met the daily recommendations of 60 min MVPA [8]. It has also been shown that prepubertal boys are more physically active than girls of the same age [9, 15, 16], and that total PA declines and sedentary time spent increases with age from childhood to adolescence [14, 16]. However, the diversity of methods used to process and score accelerometer data for youth often precludes comparison of results across studies [17]. In addition to daily MVPA level, sedentary behaviour has an important independent influence on health. Excessive sedentary time has a negative impact on body mass index (BMI), cardiovascular health, self-esteem, pro-social behaviour and academic achievement [5, 13]. In 9–15-year-old children, the prevalence rate of sedentary time ranged from 42 to 58 % of the total daily activity [18]. This suggests that it is important to reduce everyday sedentary time by increasing daily PA level.

Low levels of MVPA and high level of sedentary time have been found to be associated with higher levels of different adiposity values in children [13, 19]. Consistent evidence is still missing as to whether overweight and/or obesity influence the PA level in prepubertal children [13, 20]. Typically, normal-weight (NW) 9–11-year-old children have higher daily MVPA level than overweight (OW) children [20, 21], while no differences in daily PA level have been found between NW and OW primary school children [22]. Kettner et al. [9] reported even higher PA levels among OW 7-year-old children when compared with NW peers.

Inverse associations between different PA levels and measures of adiposity have been found in a study with 10-year-old British children [13]. Jimenez-Pavon et al. [23] demonstrated that objectively measured vigorous PA (VPA) and MVPA were negatively associated with various indices of fat mass (FM) among adolescents, but there is a lack of data about the same associations among prepubertal children. There are very few studies that have investigated the associations between objectively measured PA and sedentary time with different measures of body composition in primary school children [13]. Accordingly, further studies are needed to better clarify the associations between adiposity indices and different levels of PA in this age group.

The purpose of the present study was to determine objectively the amount of daily PA at different intensity levels and sedentary time in NW and OW 7–9-year-old children. Possible differences in PA and sedentary time between boys and girls, along with differences in adiposity were examined. In addition, the associations of objectively measured PA and sedentary time with body composition including FM and fat free mass (FFM), were examined in this specific sample of primary school children.

## Methods

### Study population

The study sample consisted of 13 randomly chosen Estonian schools all over the republic. All children from the first (aged 7–9 years) school level of selected schools and their parents received written information about the study. Four hundred and ninety-four children agreed to participate. In total, valid accelerometer data and anthropometrical measurements were obtained from 278 children (142 boys and 136 girls). Excluded children (44 % of agreed students) did not differ from those entered into the analysis in terms of gender, BMI and school level (*p* > 0.05) [24].

Written informed consents from the parent and child were obtained from all participants. The study was approved by approval 2-42 T 7, Medical Ethics Committee of the University of Tartu, Tartu, Estonia.

### Anthropometric measurements

All measurements were carried out in the school settings. Body mass and height were measured using calibrated medical digital scales (A&D Instruments, Abington, UK) and portable stadiometer (Seca 213, Hamburg, Germany) to the closest 0.05 kg and 0.1 cm, respectively, with the subject wearing light clothing without shoes. Body mass index (BMI) was calculated as body mass (kg) divided by body height squared (m^2^). Age-adjusted BMI cut-off points were used to define overweight and obese subjects [25]. Anthropometric parameters were measured according to the protocol recommended by the International Society for the Advancement of Kinanthropometry [26]. Four skinfold thicknesses (*triceps, biceps, subscapular, supra-iliac*) were measured in triplicate on the right side of the body with a Holtain caliper (Crymmych, UK) to the nearest 0.2 mm using standard procedures [26]. In every school, the same trained investigator made all skinfold thickness measurements. For all the skinfold thickness measurements, intra-observer technical errors were smaller than 1 mm and reliability greater than 95 %. Inter-observer reliability for skinfolds was higher than 90 % [27]. All measured skinfolds were also summarized (sum of skinfolds) as an indicator of total subcutaneous body fat [28]. The percentage of body fat (body fat%) and FM were calculated from *triceps* and *subscapular* skinfold thicknesses using the Slaughter et al. [29] equations. In addition, FFM in kg was derived by subtracting FM from total body mass [23]. Waist circumference was measured using a metal tape from the Centurion kit (Rosscraft, Canada) [26] and waist-to-height ratio (WHtR) was calculated as an indicator of central adiposity [30]. This index is important to identify children with high cardiometabolic risk, and a WHtR cut-point ≥0.5 is associated with increased cardiometabolic risk [31].

### Physical activity measurements

The Actigraph GT3X accelerometer (ActiGraph LLC, Pensacola, FL, USA) was used to objectively monitor whole-day PA and sedentary time. Children wore the device on a belt around the waist at the right mid-axillary line for 7 consecutive days. Children were asked to remove the device for aquatic activities. Study staff instructed children on how to wear the device. A valid recording for PA and sedentary time required at least 3 days (including at least one weekend day) of at least 10 h of wake/wear time per day [10, 32, 33]. The accelerometer data were analysed using the activity counts of 15 s epochs. For the analyses of accelerometer data, all night activity and all sequences of 20 min or more of consecutive zero counts were excluded from each individual’s recording [10, 32, 33]. Time spent sedentary was characterized by <100 counts per minute [10, 34, 35]. Activity values between 100 and 1999 counts per minute were registered as light PA (LPA) [34, 35]. The time spent in moderate PA (MPA) and VPA was calculated based upon the cut-offs of 2000 and 4000 counts per minute, respectively [28, 29]. Each individual’s accumulated PA was categorized into different intensities, and average minutes of sedentary time, LPA, MPA and VPA over measured days were subsequently calculated. Time spent in MVPA was calculated as the sum of MPA and VPA. The daily percentage of all PA intensity levels was calculated after summarizing the time spent in every intensity level including sedentary time [9]. In order to meet current PA guidelines, 60 min of MVPA was required for every single day of PA assessment [9]. For comparison, the children were considered compliant with the PA recommendations when the average MVPA over all measured days was 60 min or more [16]. PA and sedentary time levels on weekdays and weekends were examined separately and summarized to calculate average weekly PA and sedentary time as well [9]. Average measured time for both weekdays and weekend days was calculated by summarizing sedentary time and time spent in different PA intensities.

### Statistical analysis

Data analysis was made using the SPSS version 20.0 for Windows (SPSS, Inc., Chicago, IL, USA). Descriptive statistics are presented as mean and standard deviations. All variables were checked for normality before the analysis. Group differences between means were analysed with Mann–Whitney *U* test, and chi-square test was used to analyse group differences with categorical values. Differences in time spent in different PA intensities and average measured time on weekdays and weekends were analysed by paired samples *t*-test. Chi-square test was used to determine differences in percentage of time spent in different PA intensities. Multiple linear regression models were used to examine the independent associations between PA subcomponents (time spent sedentary or in LPA, MPA, VPA and MVPA) and body composition (sum of skinfolds, body fat%, WHtR, FFM) values [13, 23, 31]. The primary model (Model 1) was unadjusted. Model 2 was adjusted for age and gender [13]. The third model (Model 3) consisted of Model 2 plus FFM for body fat measures (body fat%, sum of skinfolds and WHtR) and Model 2 plus sum of skinfolds for FFM as FM and FFM are the two main compartments of human body’s composition and have classically been related [23]. In Model 4, the adjustment with sedentary time was added to Model 3 for PA intensities to examine whether the time spent in sedentary activities was independent of PA level, and the adjustment included sedentary behaviour to examine whether the associations with time spent in different PA intensities were independent of time spent sedentary [13, 31]. For sedentary time, adjustment with MVPA was applied in Model 4. The significance level was set at *p* < 0.05.

## Results

**Fig. 1:**
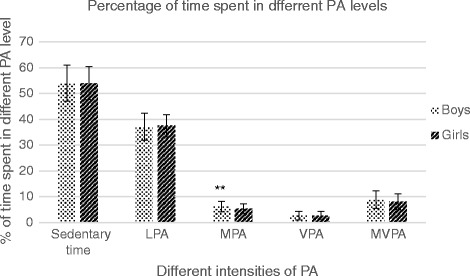
Sedentary time and different PA levels of boys and girls; values are presented mean ± SD. ** - Significantly different from boys (*p* < 0.05)

**Fig. 2:**
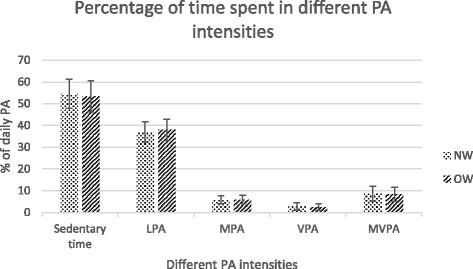
Sedentary time and different PA levels of normal-weight (NW) and overweight (OW) children; values are presented as mean ± SD

Descriptive characteristics of the study sample are shown in Table 1. There were no differences (*p* > 0.05) in age, BMI, FM and WHtR between the groups studied, while height, body mass, FFM and waist circumference were significantly higher (*p* < 0.05) in boys compared with girls. In contrast, girls had higher (*p* < 0.05) body fat % and sum of skinfolds values in comparison with boys. Almost 30 % (*n* = 83) of the children were classified as overweight or obese based on the international cut-off points [25] including 56 overweight (20.1 % of whole sample) and 27 (9.7 % of whole sample) obese children. Overall, 10.8 % (*n* = 30) of children had a WHtR of ≥ 0.5. Average wearing time of accelerometers was 804.4 ± 44.5 min/day, which did not differ between boys and girls or between NW and OW children. Almost 11 % of children were compliant with current PA recommendations. More than half of the children (60.5 %) were engaged in MVPA 60 min or more over all measured days. Boys exceeded (*p* < 0.05) girls in time spent in MPA level, while no differences (*p* > 0.05) were seen in sedentary time and other PA intensities between studied groups (Table 1; Fig. 1). Most of the accelerometer wearing time was spent being sedentary (54 %) with slightly more than one-third of wearing time spent in LPA (37 %). Only 8.5 % of the total time accounted for MVPA in which more time was spent in MPA (5.8 %) (Fig. 1). No differences (*p* > 0.05) were found in different PA intensities and time spent sedentary between NW and OW children (Fig. 2). There was a difference in activity behaviour between the weekdays and weekend days (*p* < 0.05). Higher PA levels were observed during the weekdays (Tables 2 and 3). During the weekend days, sedentary time and time spent in different PA intensities were significantly lower than during weekdays (*p* < 0.05) (Tables 2 and 3). The average measured time was significantly different between weekdays and weekend days (Tables 2 and 3). The percentage of time spent sedentary was similar in both weekdays and weekend days (Tables 2 and 3). Among boys the percentage of time spent in LPA was higher, and the proportion of time spent in MPA, VPA and MVPA was lower on weekend days than on weekdays (*p* < 0.05) (Table 2). The girls had a higher proportion of LPA on weekend days (*p* < 0.05), whereas the proportion of MPA, VPA and MVPA remained similar between weekend days and weekdays (Table 3). In total study sample, the proportion of LPA was higher on weekend days than on weekdays, and the proportion of MPA, VPA and MVPA were lower on weekend days than on weekdays (*p* < 0.05) (Table 2). Among OW children, the percentage of LPA was higher and the percentage of MPA was lower (*p* < 0.05) on weekend days compared with weekdays (Table 3).

**Table 1:** Descriptive characteristics of total sample, boys and girls

Variable	Boys (*n* = 142)	Girls (*n* = 136)	Total sample (*n* = 278)
Age (yrs)	8.0 ± 0.6	7.9 ± 0.7	7.9 ± 0.7
Height (cm)	135.1 ± 7.1	133.2 ± 6.8*	134.1 ± 6.9
Body mass (kg)	33.3 ± 8.6	30.8 ± 6.6*	32.1 ± 7.8
BMI (kg/m^2^)	18.0 ± 3.2	17.2 ± 2.6	17.6 ± 2.9
body fat%	16.9 ± 7.7	19.1 ± 5.6*	17.9 ± 6.8
FM (kg)	6.2 ± 4.6	6.1 ± 2.9	6.15 ± 3.9
FFM (kg)	27.2 ± 4.6	24.6 ± 4.3*	25.9 ± 4.6
Sum of skinfolds (mm)	35.0 ± 21.5	45.8 ± 23.2*	40.28 ± 22.9
Waist circumference (cm)	60.4 ± 8.2	57.01 ± 6.3*	58.8 ± 7.5
Waist-to-height ratio	0.45 ± 0.05	0.43 ± 0.04	0.44 ± 0.05
Sedentary time (min/day)	436 ± 62	435 ± 50	435 ± 56
Light PA (min/day)	300 ± 46	300 ± 40	300 ± 44
Moderate PA (min/day)	51 ± 17	43 ± 15*	47 ± 16
Vigorous PA (min/day)	21 ± 13	22 ± 14	22 ± 13
MVPA (min/day)	72 ± 28	65 ± 26	69 ± 27
Met current PA recommendations	13 %	9 %	11 %
Met ≥ 60 min MVPA per day (%) over all measured days	65 %	56 %	60.5 %

Values are presented as mean ± SD

*Significantly different from boys, *p* < 0.05

**Table 2:** Time and percentage of measured time spent in different PA intensities on weekdays and weekends among boys and girls

	Weekdays (min/day)	Weekend days (min/day)
Boys (*n* = 142)		
Sedentary time	450 ± 58	420 ± 77*
% of measured time	54.9 ± 6.5	54.6 ± 9
Light PA	299 ± 47	292 ± 62*
% of measured time	36.5 ± 4.9	37.8 ± 7*
Moderate PA	49 ± 15	41 ± 17*
% of measured time	6 ± 1.8	5.3 ± 2.2*
Vigorous PA	21 ± 12	18 ± 14 *
% of measured time	2.6 ± 1.4	2.3 ± 1.8*
MVPA	70 ± 25	59 ± 28*
% of measured time	8.6 ± 3	7.6 ± 3.6*
Average measured time	820 ± 53	770 ± 73*
Girls (*n* = 136)		
Sedentary time	447 ± 56	413 ± 76*
% of measured time	54.7 ± 6.9	53.8 ± 9.2
Light PA	300 ± 45	291 ± 56 *
% of measured time	36.7 ± 4.7	37.9 ± 6.3*
Moderate PA	47 ± 18	42 ± 20*
% of measured time	5.8 ± 2.1	5.5 ± 2.6
Vigorous PA	23 ± 15	21 ± 20*
% of measured time	2.8 ± 1.8	2.7 ± 2.7
MVPA	70 ± 30	63 ± 36*
% of measured time	8.6 ± 3.6	8.2 ± 4.7
Average measured time	817 ± 44	767 ± 61*
Total sample (*n* = 278)		
Sedentary time	449 ± 57	416 ± 77*
% of measured time	54.9 ± 6.7	54.2 ± 9.1
Light PA	300 ± 46	292 ± 59*
% of measured time	36.5 ± 4.8	37.9 ± 6.7*
Moderate PA	48 ± 17	42 ± 19*
% of measured time	5.9 ± 2	5.4 ± 2.4*
Vigorous PA	22 ± 14	19 ± 17*
% of measured time	2.7 ± 1.6	2.5 ± 2.3
MVPA	70 ± 27	61 ± 32*
% of measured time	8.6 ± 3.3	7.9 ± 4.2*
Average measured time	819 ± 49	765 ± 67*

Values are presented as mean ± SD

*Significantly different *p* < 0.05 from weekdays

**Table 3:** Time and percentage of measured time spent in different PA intensities on weekdays and weekends among normal-weight and over-weight children

	Weekdays (min/day)	Weekend days (min/day)
NW children (*n* = 193)		
Sedentary time	444 ± 77	411 ± 77*
% of measured time	54 ± 7	53.6 ± 9
Light PA	304 ± 46	294 ± 56*
% of measured time	37 ± 5	38.4 ± 6.3*
Moderate PA	49 ± 17	42 ± 19 *
% of measured time	6 ± 2	5.5 ± 2.6*
Vigorous PA	22 ± 14	19 ± 17*
% of measured time	2.7 ± 1.7	2.5 ± 2.4
MVPA	71 ± 28	61 ± 33*
% of measured time	8.7 ± 3.4	8 ± 4.4*
Average measured time	820 ± 47	766 ± 67*
OW children (*n* = 85)		
Sedentary time	459 ± 49	430 ± 75*
% of measured time	56.3 ± 5.9	55.6 ± 9.2
Light PA	290 ± 45	284 ± 64*
% of measured time	35.4 ± 4.5	36.6 ± 7.2
Moderate PA	47 ± 15	40 ± 17*
% of measured time	5.7 ± 1.7	5.2 ± 2.1*
Vigorous PA	22 ± 13	20 ± 17*
% of measured time	2.6 ± 1.5	2.6 ± 2.1
MVPA	68 ± 25	61 ± 30 *
% of measured time	8.3 ± 2.8	7.8 ± 3.7
Average measured time	817 ± 52	775 ± 67*

Values are presented as mean ± SD

*Significantly different *p* < 0.05 from weekdays

Independent associations between sedentary time and different PA levels with body composition values are demonstrated in Table 4. Sedentary time was not associated (*p* > 0.05) with body FM and FFM indices in unadjusted models. However, sedentary time was positively associated with indices of overall body fatness (sum of skinfolds and body fat %) and negatively with FFM, respectively, after basic adjustment for age and gender. These associations remained significant after further adjustment for FFM for overall body fatness indices, and after further adjustment for sum of skinfolds for FFM value (Table 4). Furthermore, the addition of another covariate, MVPA level, did not change independent associations between sedentary time and body composition parameters (Table 4). In addition, sedentary time was associated with the indicator of central adiposity (WHtR) after basic adjustment for age and gender, which remained significant after further adjustment for FFM and MVPA values. In unadjusted models, LPA, MPA, VPA and MVPA were significantly associated only with total adiposity values (sum of skinfolds, body fat%) but not with WHtR or FFM values. While MPA, VPA and MVPA were negatively associated with all body fat indicies (sum of skinfolds, body fat% and WHtR) and positively with FFM value, LPA was positively related to sum of skinfolds, body fat %, WHtR, as well as FFM values, after basic adjustment for age and gender. These associations remained significant after further adjustment for FFM for body fatness (sum of skinfolds, body fat% and WHtR) or sum of skinfolds for FFM value (Table 4). The further adjustment for sedentary time did not change these results.

**Table 4:** Associations in time spent in different PA intensities with body composition indicators and fat free mass

	Sum of skinfolds			Body fat percent			Waist-to-height ratio			Fat free mass		
	β	R^2^	P	β	R^2^	P	β	R^2^	P	β	R^2^	P
Sedentary time (min/day)												
Model 1	0.076	0.006	0.206	0.047	0.002	0.434	−0.023	0.001	0.701	−0.035	0.001	0.564
Model 2	0.066	0.068	<0.0001	0.042	0.034	<0.021	−0.020	0.044	<0.006	−0.059	0.170	<0.0001
Model 3	0.097	0.414	<0.0001	0.292	0.073	<0.0001	0.017	0.378	<0.0001	−0.078^a^	0.391^a^	<0.0001^a^
Model 4	−0.002	0.430	<0.0001	−0.014	0.303	<0.0001	−0.069	0.390	<0.0001	−0.053^a^	0.482^a^	<0.0001^a^
Light PA (min/day)												
Model 1	0.031	0.001	0.603	0.075	0.006	0.211	0.133	0.018	<0.025	0.048	0.002	0.428
Model 2	0.044	0.066	<0.0001	0.084	0.040	<0.01	0.134	0.061	<0.001	0.074	0.172	<0.0001
Model 3	0.004	0.405	<0.0001	0.044	0.288	<0.0001	0.087	0.385	<0.0001	0.034^a^	0.386^a^	<0.0001^a^
Model 4	0.090	0.420	<0.0001	0.132	0.303	<0.0001	0.149	0.393	<0.0001	−0.018^a^	0.480^a^	<0.0001^a^
Moderate PA (min/day)												
Model 1	−0.181	0.033	<0.002	−0.118	0.014	<0.010	−0.01	0.000	0.870	0.074	0.005	0.218
Model 2	−0.136	0.081	<0.0001	−0.085	0.040	<0.01	−0.057	0.046	<0.005	0.017	0.166	<0.0001
Model 3	−0.141	0.424	<0.0001	−0.095	0.295	<0.0001	−0.070	0.382	<0.0001	0.058^a^	0.388^a^	<0.0001^a^
Model 4	−0.134	0.424	<0.0001	−0.082	0.295	<0.0001	−0.104	0.384	<0.0001	0.049^a^	0.481^a^	<0.0001^a^
Vigorous PA (min/day)												
Model 1	−0.149	0.022	<0.013	−0.142	0.020	<0.017	−0.120	0.014	<0.043	0.009	0.000	0.875
Model 2	−0.167	0.091	<0.0001	−0.155	0.057	<0.001	−0.115	0.056	<0.001	−0.013	0.166	<0.0001
Model 3	−0.152	0.428	<0.0001	−0.148	0.308	<0.001	−0.110	0.390	<0.0001	0.063^a^	0.388^a^	<0.0001^a^
Model 4	−0.136	0.429	<0.0001	−0.146	0.308	<0.0001	−0.130	0.391	<0.0001	0.042^a^	0.481^a^	<0.0001^a^
MVPA (min/day)												
Model 1	−0.183	0.034	<0.002	−0.036	0.020	<0.018	−0.065	0.004	0.273	0.049	0.002	0.142
Model 2	−0.163	0.090	<0.0001	−0.129	0.049	<0.003	−0.091	0.051	<0.002	0.004	0.166	<0.0001
Model 3	−0.158	0.430	<0.0001	−0.129	0.303	<0.0001	−0.094	0.386	<0.0001	0.065^a^	0.389^a^	<0.0001^2^
Model 4	−0.160	0.430	<0.0001	−0.138	0.390	<0.0001	−0.140	0.390	<0.0001	0.054^a^	0.482^a^	<0.0001^a^

Model 1 – unadjusted; Model 2 – adjusted for age and gender; Model 3 – Model 2 + FFM; Model 3a for FFM: Model 2 + Sum of skinfolds; Model 4 – Model 3 + sedentary for PA intensities; Model 3 + MVPA for sedentary; Model 4a for FFM – Model 3a + sedentary for PA intensities. MVPA – moderate-to-vigorous physical activity

## Discussion

Associations between time spent at different PA levels and body composition indicators in 7–9-year-old children have still been little studied. Therefore, it is necessary to measure objectively the level of PA in children and to evaluate the influence of different PA intensities on the body fatness [13, 36], as the lack of PA and childhood obesity are already serious health problems in prepubertal children during growth [10, 36]. The main findings of our study were that although 11 % of the first level schoolchildren were compliant with current PA recommendations, 60 % of them were physically active as indicated by MVPA of 60 min or more per day over all measured days. At the same time, the children spent over half of their day being sedentary (54 %). In addition, higher MPA, VPA and consequently MVPA levels were independently associated with lower adiposity values regardless of the amount of FFM and time spent sedentary in 7–9-year-old children. These findings demonstrate that at least moderate intensity is important to influence adiposity status in children and support the suggestion that the future efforts for obesity prevention should focus on increasing daily MVPA and reducing daily sedentary time in primary school children [31].

The reported percentage (10.8 %) of our 7–9-year-old children being sufficiently physically active is smaller when compared to British [13], Spanish [10], Canadian [31], German [9] and Belgian [4] prepubertal children of similar age. For the most-cited cut-off point of 2000 cpm for MVPA [34] 37–87 % of 4–12-year-old European children could be considered physically active with regard to the current recommendations [37]. From the health perspective it is suggested to minimize the time spent in sedentary behaviours [31]. Accordingly, in addition to daily MVPA level, it is important to consider also sedentary time spent [4, 31, 38]. Too many sedentary activities among young children is a growing problem all over the world and thus there is a need to examine the sedentary behaviour habits in children. The sedentary time of primary school children has been assessed in recent studies [4, 9, 31]. Kettner et al. [9] found that 7-year-old German children spent 56.1 % of their day sedentary, whereas Spittaels et al. [4] registered that about 52 % of the day was engaged with sedentary activities in Belgian primary school children. The study with Canadian 9–11-year-old schoolchildren revealed that sedentary time accounted for 57 % of their daily activities [31]. It is interesting to note that Basterfield et al. [38] found that sedentary time performed roughly up to 80 % of the daily activities among British 7–9-year-old children. As compared with the above-mentioned results, the sedentary time of our studied Estonian primary school children studied (54 %) is similar to their peers in most other countries. The results of our study as well as other studies [4, 18, 31, 38] show that it is important to reduce sedentary time by replacing a part of sedentary time with more intensive physical activities among growing children.

In the present study, children were less active during the weekend days than during the weekdays, despite of more available time for different physical activities during the weekend days. This finding is in accordance with the results reported by Ridgers et al. [39], who considered that children appear to compensate their activity levels between days. Similar results have also been reported in German primary school children [9]. We registered significantly lower average measured time values of sedentary time and time spent in different PA intensities on weekend days than on weekdays. The children were asked to wear accelerometer during all waking hours of the study week. The children sleep more on weekend days and that was probably the reason for lower average measured time on weekend days. Therefore, it is interesting to note that during weekend days the observed children spent less time sedentary than during weekdays, but the percentage of sedentary time spent was still similar to weekdays.

Typically, it is often found that girls are more sedentary than boys [8, 9, 15]. In our study, the duration of sedentary time was equal between 7–9-year-old boys and girls. Furthermore, it is still not clearly elucidated whether prepubertal OW children spend more time sedentary than NW children. The number of OW children is growing rapidly in many countries [6, 36]. Almost 30 % of our children studied were classified as overweight or obese and 10.8 % of the children in the whole sample were obese. The number of OW children was similar to Czech and Spanish schoolchildren of the same age [10, 11] and higher than among German and British peers [9, 13]. At the same time, the study with 10-year-old Portuguese children showed that 45.8 % were overweight or obese [40]. Overweight or obesity is often considered to be associated with insufficient PA and an excess of sedentary activities [41]. The comparison of the duration of sedentary time between 7–9-year-old NW and OW children in our study showed that there were no differences as with the study of Kettner et al. [9], but it must be concluded that both groups still spent too much time in sedentary activities.

As the question remains as to whether the gender-based differences in different PA intensity levels among primary school children exist [8], the PA levels of different intensities between boys and girls were also assessed and no differences between groups in measured PA levels were found (see Fig. 1). This finding could be noted as very positive, as several studies have often revealed that boys are more physically active than girls, especially at MVPA and VPA levels already at the primary school age [9–11, 13]. This pattern of PA behaviour tends to continue among adolescents [20, 42]. The participants of our study spent on average more than 20 min per day in VPA. It has been suggested that a minimum of 15 min per day of VPA is desired to reduce the risk of developing overweight or obesity in later puberty [32]. Although primary school boys exceed girls in time spent on higher intensity PA levels in many other studies [9–11], the time spent in LPA is primarily no different between the genders [9, 43].

It is still not clear whether overweight or obesity could influence the PA of prepubertal children [17, 23]. According to the results of our study, there were no differences in time spent in measured PA intensity levels between NW and OW children. In contrast, OW primary school German children exceeded NW peers significantly in time spent in MVPA and also VPA levels [9]. However, most previous studies examining PA intensity patterns between NW and OW children of similar age demonstrate lower level of PA among OW than NW children [10, 20, 21]. In spite of fact that OW children often spend shorter periods in PA levels of higher intensities than NW children, they frequently achieve similar amount of time at LPA level [44], which is in accordance with our findings. Together with the findings of previous studies [44, 45], our results show that LPA does not decrease the values of body adiposity indicators among 7–9-year-old children.

To date, relatively few studies have examined the independent effect of overall sedentary time and PA levels on body composition indicators among primary school children [31]. In our study, we found that sedentary time was positively associated with indices of overall body fatness (sum of skinfolds, body fat %) and negatively associated with FFM after adjustment for several confounders (see Table 4). This is in contrast with previous findings, which have shown that associations between accelerometer-assessed sedentary time and adiposity in 8–11-year-old children are often not present or disappear after adjusting for MVPA [36]. Recent investigations have shown that WHtR, which is an indicator of central adiposity, is a useful index to identify children with high cardiometabolic risk, whereas a WHtR cut-point of ≥0.5 is associated with increased cardiometabolic risk [30]. Sedentary time was also independently associated with WHtR after basic adjustment for age and gender, and also after further adjustment for FFM and MVPA values in our children. These results agree with previous investigations in adults showing that objectively measured sedentary time is related to higher adiposity and/or poorer cardiometabolic health independent of MVPA [4]. In their experiment with 8–11-year-old children, Hjorth et al. [36] found that sedentary time was positively associated with fat mass index, which was used as an indicator of body fatness. The above-mentioned findings strongly suggest that decreasing sedentary time among children is important in achieving a healthy body composition during growth in primary school children.

The specific role of objectively measured PA levels on FFM as an indicator of muscle compartment is still little studied in elementary school children [23, 42]. It is demonstrated that children whose VPA levels are higher tend to gain less weight over time and that VPA is the component of total PA that appears most strongly associated with different indices of adiposity [1, 13, 32]. The findings of our study suggest that MPA, VPA and consequently MVPA were negatively associated with indices of adiposity and positively with FFM independent of each other. Similarly to our results, VPA was positively associated with FFM after adjusting for several confounders such as FM, age and gender in the study of Jiménez-Pavón et al. [23] of 14–15-year-old adolescents. It has been suggested that MPA and MVPA with longer duration has a greater effect on muscular component of the body of a growing child [46], while PA could negatively influence FM by increasing total energy expenditure and PA at higher intensity levels can be conducive to influence muscle mass [23]. It is also suggested that the increase in muscle mass has an additional effect on total energy expenditure due to its own metabolic requirements [46]. However, these assumptions should be confirmed in future studies.

The present study has some limitations. The observed associations cannot be interpreted to reflect causal relationships due to the cross-sectional design of the study. In addition, socio-economic status (SES) and the sedentary time pursuits of study participants were not assessed, although SES has been reported to influence the PA and sedentary time in children [23]. However, similarly to our study, socio-economic status has not also been measured in other studies that have objectively measured PA and sedentary time in children [8, 10, 32, 39]. In addition, body composition was measured by indirect anthropometric method using skinfolds [29], although this method has been validated as appropriate for this purpose [29, 47]. The way we estimated FFM as a marker of muscle mass could also include some bias as FFM includes also bone and residual mass [47]. However, the strengths of our study are a sample selected from 13 schools from different counties of Estonia covering all areas within a country, the use of accelerometers to objectively measure PA, and the use of several covariates including body composition and objectively measured PA values when analysing independent associations between PA and body composition variables.

## Conclusion

The present study shows that MVPA and VPA levels have an important effect on indices of adiposity and the muscular component. Simultaneously, sedentary time was positively associated with adiposity indices independent of several confounders among primary school children. The children were less active during the weekend days than during the weekdays, but they were less sedentary on weekend days compared with weekdays. Further studies are needed to examine the effect of PA intervention programmes on the body composition of children.

## Availability of data and materials

The raw data in excel file under de-identification policy could be provided via the e-mail of corresponding author upon request for research purpose only.

## Abbreviations

BMI: body mass index
body fat %: percentage of body fat
cm: centimetre
FFM: fat free mass
FM: fat mass
h: hour
kg: kilogram
LPA: light physical activity
m^2^: square metre
mm: millimetre
MPA: moderate physical activity
MVPA: moderate-to-vigorous physical activity
NW: normal-weight
OW: normal-weight
PA: physical activity
s: second
SD: standard deviation
VPA: vigorous physical activity
WHtR: waist-to-height ratio
